# The investigation of the association of pregnancy weight gain on maternal and neonatal gut microbiota composition and abundance using 16sRNA sequencing

**DOI:** 10.1186/s12884-022-05289-4

**Published:** 2023-02-13

**Authors:** Zhiying Song, Hui Liu

**Affiliations:** 1 Department of obstetrics, Children’s Hospital of Shanxi (Women’s Health Centre of Shanxi), No.13, Xinmin North Street, Taiyuan, 030000 Xinghualing District, Shanxi Province China; 2 Shanxi Medical University, NO. 56, Xinjian South Street, Taiyuan, Yingze District, Shanxi Province China

**Keywords:** Pre-pregnancy body mass index, Pregnancy weight gain, Gut microbiota, Pregnant women, Newborns

## Abstract

**Objective:**

To investigate the association of pregnancy weight gain on gut microbiota in pregnant women and newborns.

**Methods:**

Pregnant women who had regular antenatal check-ups and were hospitalised for delivery at Shanxi Maternal and Child Health Hospital from September 2020 to December 2020 were selected as the study subjects. They were divided into the normal pre-pregnancy weight-normal pregnancy weight gain group (N-NG group), the normal pregnancy weight-excessive pregnancy weight gain group (N-EG group), the pre-pregnancy overweight/obese-normal pregnancy weight gain group (O-NG group) and the pre-pregnancy overweight/obese-excessive pregnancy weight gain group (O-EG group). Faecal samples of the pregnant women before delivery (37–41^+ 6^ weeks of gestation) and the first meconium samples of their newborns were collected, sequenced for 16S rRNA gut microbiota and analysed. The results of different gut microbiota were compared separately. χ2 test, a one-way analysis of variance or the rank sum test were performed according to data type and distribution. The differences in the Alpha diversity between the groups were analysed using the Kruskal–Wallis rank sum test. The differences in the Beta diversity between the groups were analysed using the Adonis method.

**Results:**

A total of 126 pre-delivery faecal samples from pregnant women and the first faecal samples from their newborns were collected. Seven species with significant abundance differences between the maternal O-NG and N-EG groups and 27 species with significant abundance differences in the newborns were analysed by LEfSe. In the Alpha diversity analysis, the differences in the maternal observed species index and the Chao1 index were statistically significant (*p* < 0.05) when compared between the groups (O-EG group versus the O-NG group, N-EG group and N-NG group), and the differences in the Shannon index and Simpson index were not statistically significant (*p* > 0.05) when compared between the groups. The neonatal observed species index, Chao1 index, Shannon index and Simpson index showed statistically significant differences in the comparison between the N-EG and O-EG groups (*p* < 0.05). In the Beta diversity analysis, the maternal samples did not differ Significantly between the four groups (*p* > 0.05), while the neonatal samples differed Significantly between the N-EG and N-NG, O-NG, and O-EG groups (*p* < 0.05).

**Conclusion:**

Pregnancy weight gain affects the composition and abundance of maternal and neonatal gut microbiota species as well as the diversity of neonatal gut microbiota.

## Key messages

Some studies have not found that weight gain during pregnancy influences gut microbiota in infants under two years of age.

Gut microbiota dysbiosis and metabolic disorders often occur in maternal obesity during pregnancy, which also affect the transmission of the maternal flora to their offspring and further lead to metabolic disorders in the offspring.

Pregnancy weight gain affects the composition and abundance of maternal and neonatal gut microbiota species and the diversity of neonatal gut microbiota.

## Introduction

In recent years, the pregnancy body mass index (PBMI) of women of reproductive age has been on the rise [1]. A pre-pregnancy classification of being overweight or obese and excessive gestational weight gain (EGWG) increase the risk of adverse pregnancy outcomes such as gestational diabetes, gestational hypertensive disorders, caesarean section, preterm delivery, over-gestational age foetuses and macrosomia [2]. As the second genome in the body, the gut microbiota provides genetic diversity and plays an important role in host nutrient metabolism, maintenance of the structural integrity of the intestinal mucosal barrier and immune regulation [3], all of which are important factors in the pathogenesis of several diseases. In recent years, bacterial DNA has been reported to be present in the cord blood, placenta, amniotic fluid, and meconium from healthy pregnancies, suggesting that the initial exposure of the human body to microorganisms may be before birth, when microorganisms are acquired from the mother and the maternal microbiota plays an important role in the colonisation of the offspring microbiota [4]. The bacterial 16S rRNA gene has a spaced arrangement of the conserved regions and variable regions, of which the variable regions are generally species-specific and can reflect the distance of the genetic relationship between bacteria, and the taxonomic characteristics of each bacterium can be obtained by analysing the sequences of the variable regions. Therefore, the taxonomic identification and precise quantification of mixed strains in complex samples can be achieved by combining the advantages of high-throughput sequencing technology and the strain identification of the 16S rRNA gene [5].

Maternal obesity during pregnancy often occurs with gut microbiota dysbiosis and metabolic disorders. This affects the transmission of the maternal flora to the offspring and leads to metabolic disorders in the offspring [6]. Gut microbiota composition is closely related to body fat, and gut microbiota can participate in fat synthesis and energy stores by increasing dietary absorption of energy, changing the duration of intestinal digestion and absorption, or by regulating different signaling pathways. Obese subjects have abnormal gut microbiota composition and short-chain fatty acids (SCFAs) metabolism [7]. Found a significant difference in the structure of distal colonic bacteria between obese and non-obese people. Pregnancy is an ideal model to assess obesity and weight gain. Physiological, biochemical, and metabolic changes in tissues and organs are evident during pregnancy in women, and these changes are accompanied by changes in colonizing bacteria. The gut microbiota of older pregnant women are significantly different from those of women of normal reproductive age, and the diversity of intestinal bacteria decreases with the increase; more Firmicutes and Clostridium are present in the first trimester, and Enterobacteriaceae and Streptococci increase in the third trimester [8]. Abnormalities in intestinal bacteria during pregnancy can affect maternal-fetal metabolism through SCFA [9] In this study, the association of pregnancy weight gain on the gut flora characteristics of pregnant women and newborns is observed.

## Materials and methods

### Research subjects

Pregnant women with regular antenatal check-ups and hospital deliveries at the Shanxi Maternal and Child Health Hospital from September 2020 to December 2020 were recruited, including a total of 58 pregnant women and 68 newborns (of which 58 were paired with the pregnant women). Maternal weight and height are obtained through regular measurement (biweekly) in the hospital. According to the pre-pregnancy body mass index and the pregnancy weight gain criteria, they were divided into four groups: normal pre-pregnancy weight-normal pregnancy weight gain group (N-NG group), normal pregnancy weight-excessive pregnancy weight gain group (N-EG group), overweight/obese pre-pregnancy-normal pregnancy weight gain group (O-NG group) and overweight/obese pre-pregnancy-excessive pregnancy weight gain group (O-EG group). In this study, gestational weight gain (GWG) was calculated as weight at term minus pre-pregnancy weight, and all maternal feces were collected at 37–42 weeks’ gestation. The criteria for excessive weight gain during pregnancy were calculated according to the 2009 American Institute of Medicine regulations. The normal weight gain range during pregnancy was 11.5–16 kg, 7–11.5 kg and 5–9 kg in normal weight, overweight and obesity pregnant women, respectively. The excessive weight gain during pregnancy was > 16 kg, > 11.5 kg and > 9 kg in normal weight, overweight and obesity pregnant women, respectively. There were 18 pregnant women and 20 newborns in the N-NG group, 20 pregnant women and 20 newborns in the N-EG group, 9 pregnant women and 13 newborns in the O-NG group, and 11 pregnant women and 15 newborns in the O-EG group.

Diagnostic criteria: Pre-pregnancy overweight and obesity diagnostic criteria referred to the 2003 edition of the ‘Guidelines for the Prevention and Control of Overweight and Obesity in Chinese Adults (Trial)’; a PBMI ≥24 kg/m^2^ is considered high. Normal: 18.5 kg/m^2^ ≤ BMI < 24 kg/m^2^_._ Overweight: 24 kg/m^2^ ≤ BMI < 28 kg/ m^2^_._ Obesity: BMI ≥28 kg/m^2^_._

According to the weight gain criteria during pregnancy specified by the American Institute of Medicine in 2009, EGWG was diagnosed by exceeding the recommended criteria. Inclusion criteria: maternal age of 25–40 years old, natural conception, singleton pregnancy, and 37–41^+ 6^ weeks of gestation; no microecological samples taken during pregnancy, no antibiotics used 1 month before specimen collection; and no signs of infection or potential factors for infection during prenatal and delivery. Exclusion criteria: BMI < 18.5 kg/m^2^; chronic underlying diseases (including diabetes mellitus, rheumatoid arthritis, chronic infectious diseases, gastrointestinal diseases, and cardiovascular diseases) before pregnancy; premature rupture of foetal membranes; and anxiety and depression.

### Specimen collection and Flora testing

The pregnant women were given 40 ml sterile PS stool collection cups and sterile gloves in advance to collect their stool before delivery along with the first faeces of the newborn after birth. At least 2 g of the central part of the stool was collected with a spoon in the stool collection cup. Each sample was divided into 1.5-mL sterile EP tubes, numbered and frozen in the laboratory at − 80 °C within 2 h. The total meconium DNA was extracted and used as a template for polymerase chain reaction amplification and quantification under the action of the 16S V3–V4 region specific amplification primers. The target bands were recovered, and the final 16S V3–V4 region library was constructed. The reads were clustered and classified into operational taxonomic units (OTUs) according to the 97% similarity level. The sequences of the OTUs were compared with existing microbial databases, annotated and analysed by bioinformatics to obtain the diversity indices and species information. Subsequently, the diversity of the flora between different groups was analyzed for Alpha diversity and Beta diversity.

### Statistical methods

Statistical analyses were performed using IBM SPSS 26.0 and R 3.4.4 with a test level of α = 0.05. The measurement data were expressed as the mean ± standard deviation($\overline{\textrm{x}}$ ± SD), and the count data were expressed as number of cases (%).

The χ2 test, a one-way analysis of variance or the rank sum test was performed for the basic characteristics of the pregnant women and the newborns between different pregnancy weight gain groups according to data type and distribution. The software Usearch (version 10) was used to classify all sequences into OTUs, and the OTUs at the 97% similarity level were analysed for bioinformatic statistics. The gut microbiota Alpha diversity was evaluated using the Shannon index, Simpson index, Chao1 index and observed species index. The differences in the Alpha diversity between the groups were analysed using the Kruskal–Wallis rank sum test. The differences in the Beta diversity between the groups were analysed using the Adonis method. SPSS offers Bonferroni-adjusted significance tests for pairwise comparisons. This adjustment is available as an option for post hoc tests and for the estimated marginal means feature. First, divide the desired alpha-level by the number of comparisons. Second, use the number so calculated as the *p*-value for determining significance. NMDS were used at the OTU level to compare the differences in the Beta diversity of the microflora between the different weight gain groups.

## Results

### Comparison of general clinical data characteristics of the four groups

There were no statistically significant differences between the groups in gestational age, delivery gestational week, number of pregnancies and number of births in each group (*p* > 0.05). There were no statistically significant differences in maternal pregnancy age, birth history, gestational age, birth weight and birth mode of the newborns between the groups (*p* > 0.05). This indicates that the groups were comparable (see Tables 1 and 2).

**Table 1:** Comparison of the general data between the four groups of pregnant women($\overline{\textrm{x}}$ ± S)/n(%)

	N-NG(*n* = 18)	N-EG(*n* = 20)	O-NG(*n* = 9)	O-EG(*n* = 11)	F/χ^2^	*P*
Age (age)	30.76 ± 3.37	30.35 ± 4.92	32.78 ± 4.66	31.00 ± 4.03	0.068	0.567
Birth for gestational age (days)	274.39 ± 5.51	278.55 ± 5.79	273.78 ± 7.07	272.36 ± 9.23	2.558	0.065
Childbearing history						
Primiparity	9 (50.0)	3 (70.0)	3 (33.5)	3 (27.3)	6.332^*^	0.097^*^
Multiparity	9 (50.0)	6 (30.0)	6 (66.7)	8 (72.7)		

*N-NG *the normal pre-pregnancy weight-normal pregnancy weight gain group, *N-EG *the normal pregnancy weight-excessive pregnancy weight gain group, *O-NG *the pre-pregnancy overweight/obese-normal pregnancy weight gain group, *O-EG *the pre-pregnancy overweight/obese-excessive pregnancy weight gain group, *F *For the Fisher exact test, χ^2^ value

**Table 2:** Comparison of the general data between the four neonatal groups($\overline{\textrm{x}}$ ± S)/n(%)

	N-NG(*n* = 20)	N-EG(*n* = 20)	O-NG(*n* = 13)	O-EG(*n* = 15)	F/χ^2^	*P*
Maternal age (Age))	30.65 ± 4.59	30.30 ± 5.41	32.69 ± 3.73	30.60 ± 3.38	0.845	0.475
Fetal age (day)	276.55 ± 5.32	277.50 ± 7.45	274.92 ± 6.33	277.87 ± 8.95	0.494	0.688
Birth weight(g)	3257.00 ± 292.20	3389.00 ± 335.43	3235.00 ± 466.35	3568.00 ± 428.94	3.578	0.061
Childbearing history					4.523	0.210
Primiparity	11 (55)	13 (65)	4 (30.8)	6 (40)		
Multiparity	9 (45)	7 (35)	9 (69.2)	9 (60)		
The way of birth					7.141	0.068
C-sect	7 (35)	13 (65)	10 (76.9)	10 (66.7)		
Eutocia	13 (65)	7 (35)	3 (23.1)	5 (33.3)		
Sex					0.309	0.958
Female	9 (45)	9 (45)	6 (46.2)	8 (53.3)		
Male	11 (55)	11 (55)	7 (53.8)	7 (46.7)		

*N-NG *the normal pre-pregnancy weight-normal pregnancy weight gain group, *N-EG *the normal pregnancy weight-excessive pregnancy weight gain group, *O-NG *the pre-pregnancy overweight/obese-normal pregnancy weight gain group, *O-EG *the pre-pregnancy overweight/obese-excessive pregnancy weight gain group, *F *For the Fisher exact test, χ^2^ value

### OTU level analysis

A total of 126 samples from the four groups of mothers and newborns yielded a total of 5,783,854 sequenced entries of optimised data. The mean value of the sequencing depth was 0.998332.

This study performed OTU clustering on non-repeated sequences (excluding single sequences) according to 97% similarity, and chimeras were removed during clustering to obtain representative sequences of OTUs. The total number of OTUs in the four groups of pregnant women was 1468, and the total number of OTUs in the four groups of newborns was 3514. The similarity and overlap of the OTU number and composition of each group can be visualised in a Venn diagram. The total number of OTUs in the four groups of pregnant women was 403, the number of OTUs unique to the N-NG group was 119, the number of OTUs unique to the N-EG group was 113, the number of OTUs unique to the O-NG group was 183 and the number of OTUs unique to the O-EG group was 72*.* The total number of OTUs in the four neonatal groups was 720, of which 393 OTUs were unique to the N-NG group,720 OTUs were unique to the N-EG group, 46 OTUs were unique to the O-NG group, and 49 OTUs were unique to the O-EG group.

### Species composition analysis

The proportion of annotations for each taxonomic level of OTUs was counted to obtain the relative abundance of each species at each taxonomic level.

At the phylum taxonomic level, all four groups of were dominated by *Firmicutes*, *Actinobacteria*, *Bacteroidetes*, and *Proteobacteria*. The proportions of *Firmicutes* in the four groups were 62.90, 66.32, 59.60, and 65.09%, respectively. The proportions of *Actinobacteria* were 16.94, 16.20, 17.12, and 12.85%, respectively. The proportions of *Bacteroidetes* were 13.26, 13.25, 12.09, and 9.28%, respectively, and the proportions of *Proteobacteria* were 4.62, 4.61, 10.69, and 12.40%, respectively. The abundance of *Proteobacteria* was higher in the O-NG and O-EG groups than in the N-NG and N-EG groups (10.69 and 12.40% versus 4.62 and 4.61%), and the difference between the four groups was not statistically significant per the Kruskal–Wallis rank sum test (*p* > 0.05).

At the phylum taxonomic level, all four groups were dominated by *Proteobacteria*, *Bacteroidetes*, *Firmicutes*, and *Actinobacteria*. The proportions of *Proteobacteria* in the four groups were 55.21, 55.23, 47.96 and 52.86%. The proportions of *Bacteroidetes* were 28.14, 18.61, 29.35 and 24.52%. The proportions of *Firmicutes* were 6.34, 17.92, 8.61 and 14.64%. The proportions of *Actinobacteria* were 6.94, 5.27, 9.92 and 5.84%. The relative abundance of *Firmicutes* was higher in the EGWE group (the N-EG and O-EG groups combined) than in the normal gestational weight gain (GWG) group (the N-NG and O-NG groups combined), while *Bacteroidetes* and *Actinobacteria* were lower in the EGWE group than in the normal GWG group, and the differences were statistically significant (*p* < 0.05).

### Alpha diversity analysis

**Fig. 1:**
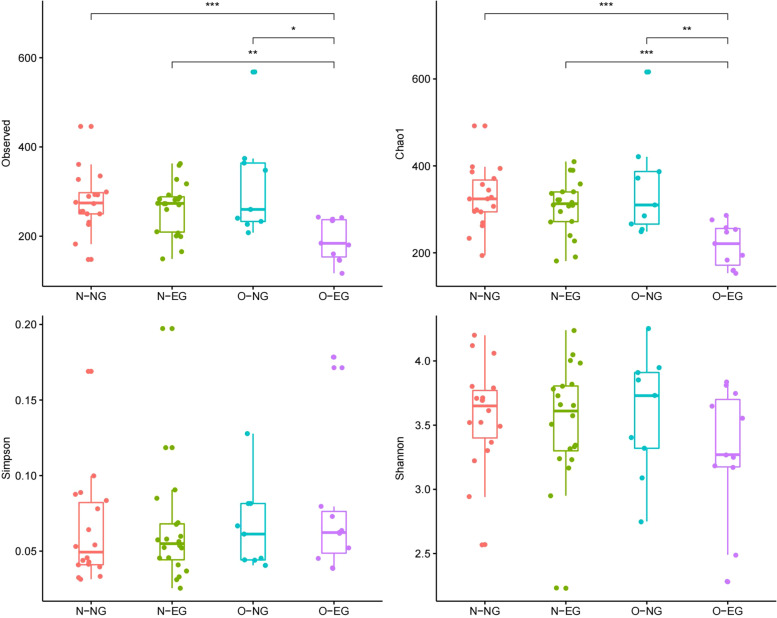
The clockwise direction shows the differences between groups: observed_species index, chao1 index, shannon index and simpson index (the maternal group). By one-way ANOVA analysis, *: *p* < 0.05, **: *p* < 0.01, ***: *p* < 0.001

In the maternal group, as is shown in Fig. 1, this study found no statistically significant differences between the groups in the Shannon and Simpson indexes (*P* > 0.05); however, there were statistically significant differences between the groups in the Chao1 and observed species indexes (O-EG group versus O-NG group, N-EG group and N-NG group) (*P* < 0.05). This result indicates that if only species abundance is considered, it can be concluded that in high PBMI, the species abundance of the EGWG group is less than that of the normal GWG group (O-EG group versus O-NG group); when EGWG occurred, the species abundance of the high PBMI group is less than that of the normal PBMI group (O-EG group versus N-EG group).

**Fig. 2:**
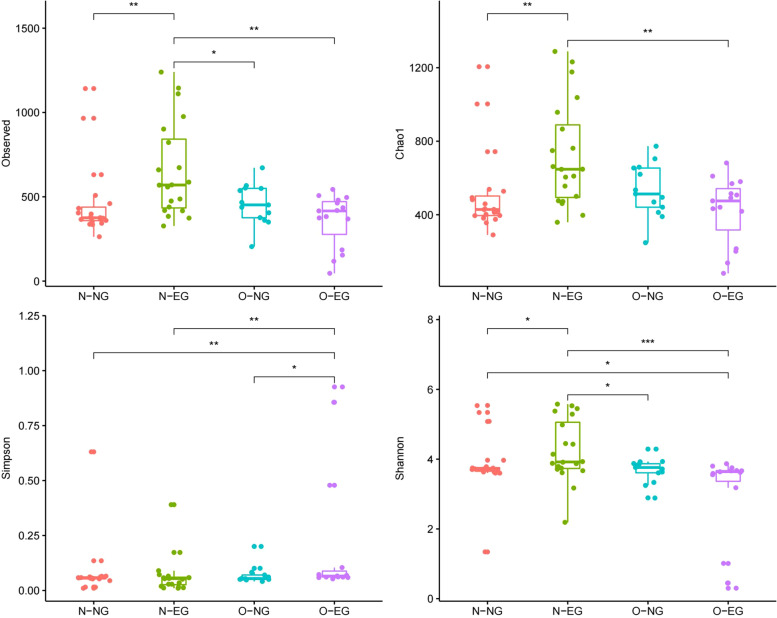
The clockwise direction shows the differences between groups: observed_species index, chao1 index, shannon index and simpson index (the neonatal group). By one-way ANOVA analysis, *: *p* < 0.05, **: *p* < 0.01, ***: *p* < 0.001

In the neonatal group, as is shown in Fig. 2, this study found statistically significant differences between the groups in the Shannon index, Simpson index, Chao1 index, and observed species index (*p* < 0.05). The differences in all four Alpha diversity indices were statistically significant at the same time only when the N-EG group and O-EG group were compared. This indicates that the Alpha diversity of the high PBMI group is lower than that of the normal PBMI group when EGWG occurred. Similarly, the differences in all four indices of Alpha diversity were not statistically significant when comparing the N-NG and O-NG groups, indicating that the differences in Alpha diversity between the high PBMI group and the PBMI normal group were not statistically significant in the normal GWG group.

### Beta diversity analysis

**Fig. 3:**
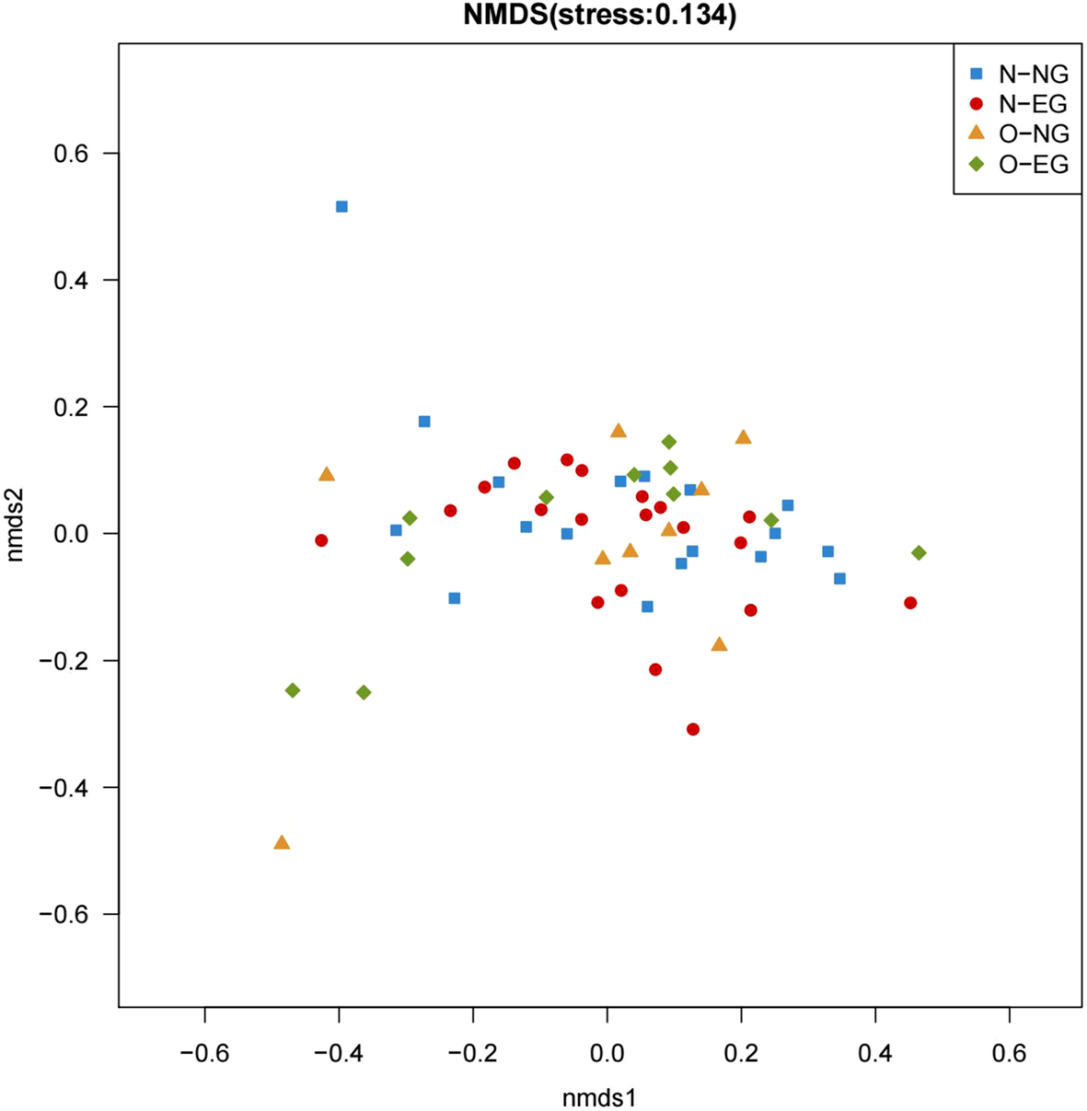
NMDS diagram of pregnant women (Weighted UniFrac). NMDS:Non-metric multidimensional scaling

In the maternal group, as can be seen from the NMDS plot (Fig. 3), the groups of maternal samples cannot be clearly distinguished from one another. Meanwhile, the results of the Adonis analysis based on weighted UniFrac showed no statistically significant differences in the microbial community structure between the four groups (*R*^*2*^ = 0.033, *p* > 0.05).

**Fig. 4:**
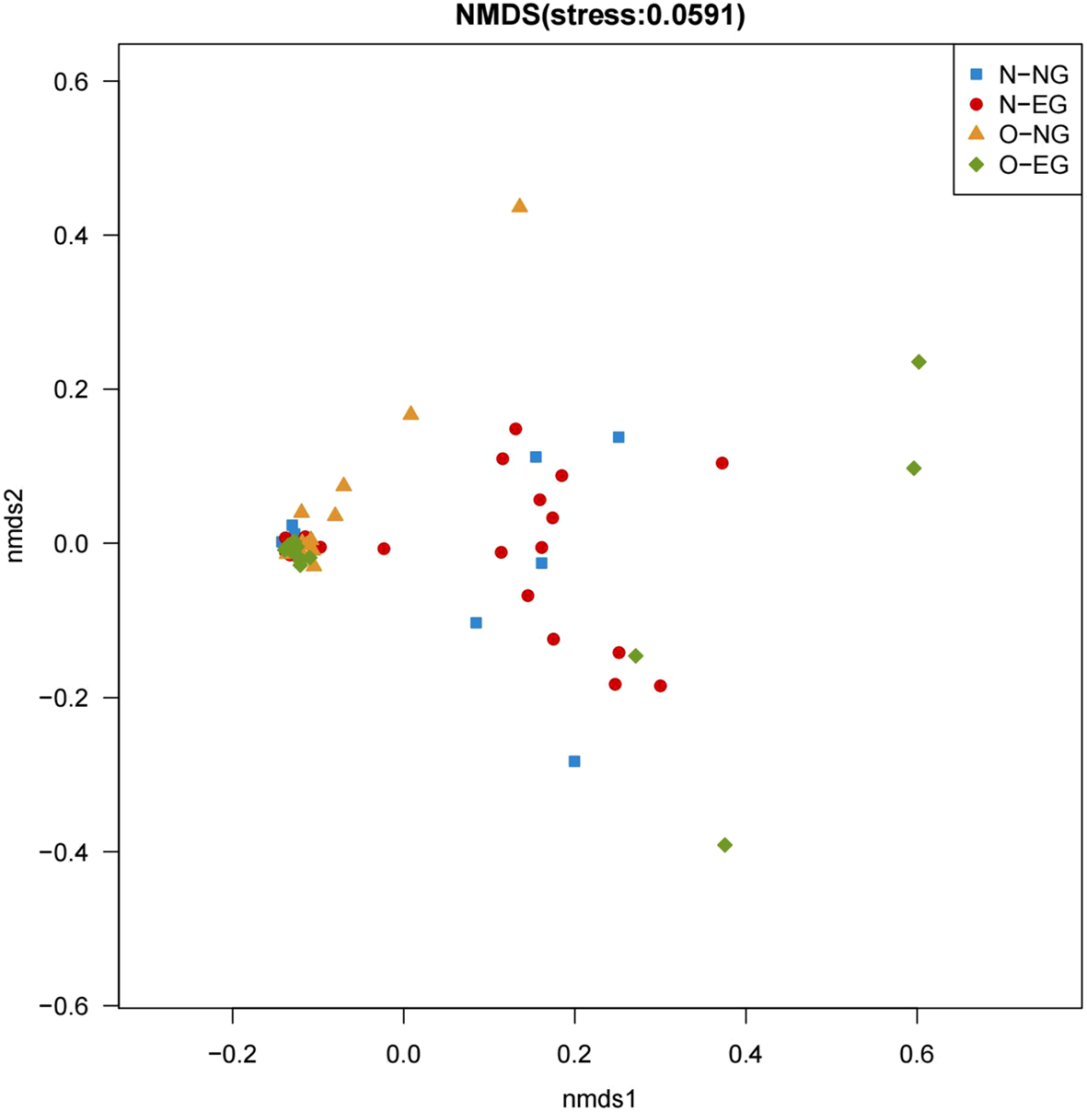
neonatal NMDS diagram (Weighted UniFrac). NMDS:Non-metric multidimensional scaling

In the neonatal group, as can be seen from the NMDS plot (Fig. 4), the neonatal samples in the N-EG group were clearly distinguishable from the other three groups, while the other three groups were indistinguishable. The results of the Adonis analysis showed statistically significant differences in the microbial community structure between the four groups (*R*^*2*^ = 0.136, *p* < 0.05), and the differences between the N-EG group and the other three groups were found to be significant after pairwise comparisons (*p* < 0.05). When these results were combined with the NMDS plot analysis, the results demonstrated that the difference in intestinal community structure between the EGWG and normal GWG groups was significant at normal PBMI, and the difference in intestinal community structure between the normal PBMI and high PBMI groups was significant in the EGWG group.

### LEfSe variance analysis

**Fig. 5:**
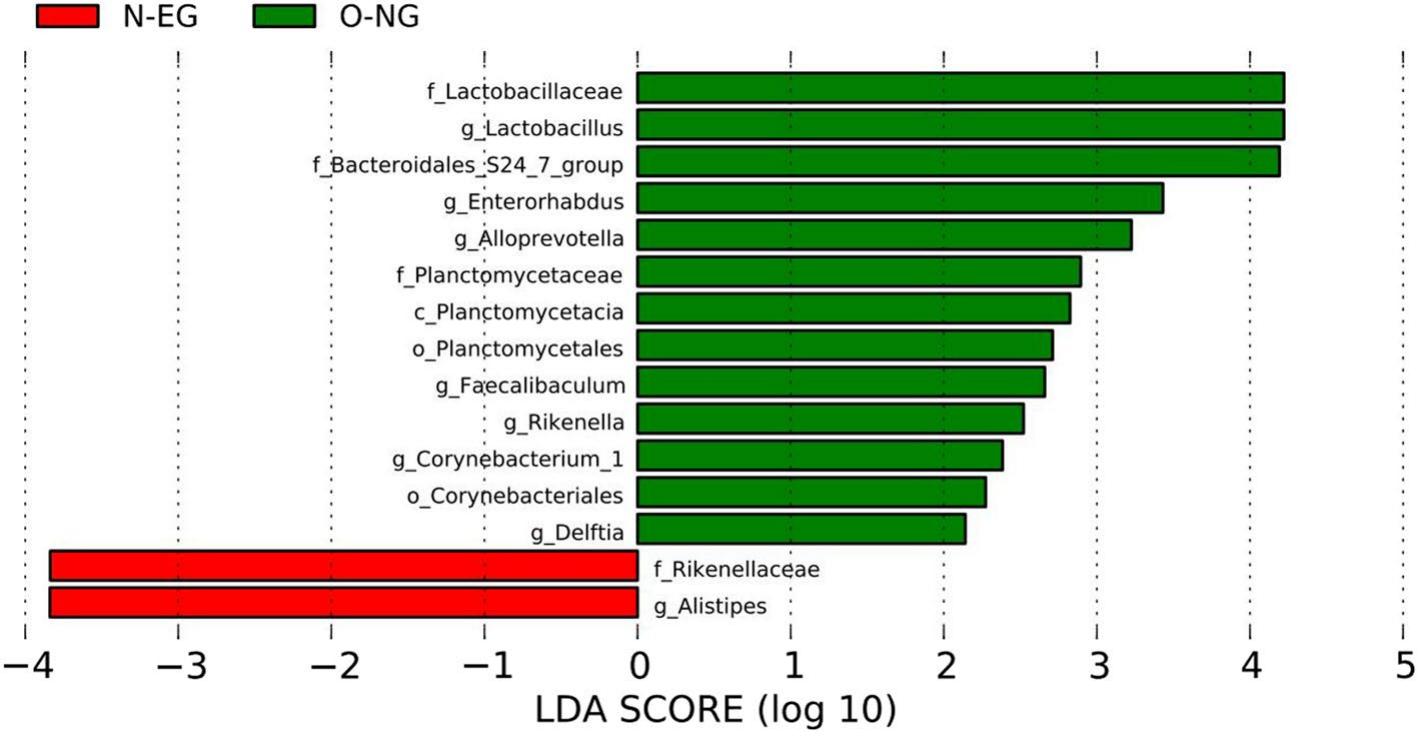
Bar graph of LDA for LEfSe analysis of pregnant women. LDA: Linear discriminant analysis; LEfSe: LDA Effect Size Analysis.

In the maternal group, the LEfSe analysis showed (Fig. 5) that there were no species with significant differences in abundance between the O-EG and N-NG groups. *Lactobacillaceae*, *Lactobacillus*, *Bacteroidales* S24–7, *Enterorhabdus*, *Alloprevotella*, *Planctomycetaceae*, *Planctomycetacia*, *Planctomycetales*, *Faecalibaculum*, *Corynebacterium*, *Corynebacteriales* and *Delftia* were significantly increased in the O-NG group. *Rikenellaceae* and *Alistipes* were significantly increased in the N-EG group.

**Fig. 6:**
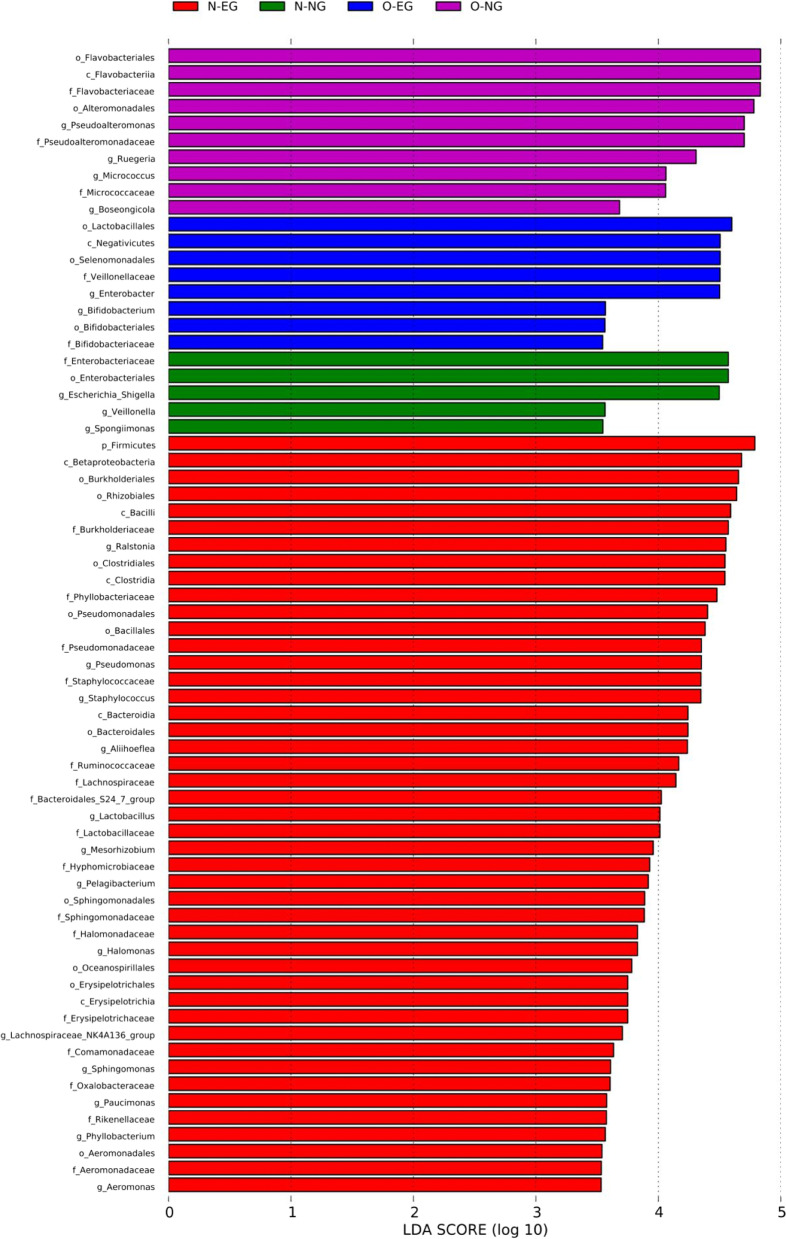
Bar graph of LDA for neonatal LEfSe analysis. LDA: Linear discriminant analysis; LEfSe: LDA Effect Size Analysis. Note: N-NG: the normal pre-pregnancy weight-normal pregnancy weight gain group, N-EG: the normal pregnancy weight-excessive pregnancy weight gain group, O-NG: the pre-pregnancy overweight/obese-normal pregnancy weight gain group, O-EG: the pre-pregnancy overweight/obese-excessive pregnancy weight gain group

In the neonatal group, *Flavobacteriales*, *Flavobacteriia*, *Flavobacteriaceae*, *Alteromonadales*, *Pseudoalteromonadaceae* and *Micrococcaceae* were biomarkers of the O-NG group. Lactobacillales, *Selenomonadales*, *Veillonellaceae*, *Enterobacter*, *Bifidobacteriales* and *Bifidobacteriaceae* were biomarkers of the O-EG group. *Enterobacteriaceae*, *Enterobacteriales* and *Veillonella* were biomarkers of the N-NG group. *Betaproteobacteria*, *Rhizobiales*, *Bacilli*, *Clostridiales*, *Clostridia*, *Phyllobiiaceae*, *Pseudomonadales*, *Pseudomonadaceae*, *Bacillales*, *Staphylococcus*, *Bacteroidia*, *Bacteroidales*, *Ruminococcaceae*, *Bacteroidaceae* S24–7, *Lactobacillus*, *Lactobacillus*, *Sphingomonadales*, *Sphingomonadales*, *Erysipelnotrichales*, *Erysipelotrichia*, *Erysipelotrichaceae*, *Lachnospiraceae*, *Lachnospiraceae*_NK4A136, *Rikenomycetes*, *Phyllobacterium*, *Aeromonadales*, *Aeromonadaceae* and *Aeromonas* were biomarkers of the N-EG group. See Fig. 6.

## Discussion

In healthy humans, most species isolated from the gut microbiota belong to four phyla: *Firmicutes* (60–65%), Bacteroidetes (20–25%), *Proteobacteria* (5–10%), and *Actinobacteria* (3%), which together constitute more than 97% of the intestinal microbial population [7]. In this study, the dominant phylum in the maternal and neonatal groups were the same four species mentioned above. In the mothers, the *Firmicutes* and the *Actinobacteria* accounted for about 80%, and in the neonates, the *Proteobacteria* and the *Bacteroidetes* accounted for about 90%.

The LEfSe analysis of the maternal and neonatal gut microbiota in the four groups revealed that *Lactobacillus*, *Bacteroidales S24–7*, *Enterorhabdus, Alloprevotella*, *Faecalibaculum* and *Rikenellaceae* were significantly different in abundance in the overweight/obese and normal GWG maternal groups before pregnancy. In neonates, the results indicated more aerotolerant anaerobes in the N-EG group among species with significant differences in abundance compared with the other three groups. It was shown that one of the associations of maternal pre-pregnancy obesity and EGWG was an increase in aerotolerant anaerobes (e.g. *Enterobacteriaceae*, *Enterococcus*, *Streptococcus*, Staphylococcus, *Escherichia coli*, *Enterococcus faecalis*, *Klebsiella*, *Aeromonas*, *Pseudomonas* and *Acinetobacter*) in the gastrointestinal microbiota of the offspring. Aerotolerant anaerobes are normal in the early colonisation of the neonatal intestine, and the consumption of available oxygen by these early colonised aerotolerant anaerobes promotes the establishment of obligate anaerobes (e.g. *Bifidobacterium*, *Bacteroidales*, *Veillonella*, and *Ruminococcus*) [8, 9]. However, the excessive increase of aerotolerant anaerobes in the gut of newborns in the presence of maternal obesity may instead make it possible that the maturation of the gut microbiota (usually occurring in the first 2 years of childhood) will be prevented or delayed, as aerotolerant anaerobes do not give way to the obligate anaerobes normally present in the adult gut [10].

The relationship between maternal gestational weight gain and neonatal flora diversity has been minimally studied, and the findings are inconsistent. The study by Mueller et al. [11] showed that the Beta diversity of the faecal microbiota in normal birth neonates was associated with the mother’s being overweight. Collado et al. [12] found that the number of *Bacteroidete* (i.e. *Prevotella*) was lower and the number of *Clostridium histolyticum* was higher in the overweight maternal group compared with the infants in the underweight maternal pregnancy group 1 month after birth. In contrast, the effect of maternal pregnancy weight gain on the gut microbiota of infants up to 2 years of age was not found in the study by Stanislawski et al. [13] The inconsistent results of these studies may be related to the rapid changes in the gut microbiota of infants and children until about 2 years of age when it gradually stabilises [14], and factors such as breastfeeding, timing of stool sampling, and postnatal environment may affect the colonisation of the infant’s gut microbiota. There was a higher abundance of species composition in newborns in the pre-pregnancy normal body weight group in this study. In addition, in this study, the differences in the relative abundance among the groups in the neonatal group were statistically significant for *Bacteroidetes*, *Firmicutes*, and *Actinobacteria*. In the EGWG neonatal group, *Actinobacteria* and *Bacteroidetes* were lower than in the normal GWG group and *Firmicutes* were higher than in the normal GWG group. In terms of flora diversity, this study found statistically significant differences in the Beta diversity between the PBMI and GWG subgroups (N-EG group versus N-NG group; N-EG group versus O-EG group). This study’s results indicated that neonatal flora Alpha diversity in the pre-pregnancy overweight/obese group was lower when compared with the pre-pregnancy normal body group when maternal EGWG occurred.

Gut microbiota composition is closely related to body fat, and gut microbiota can be involved in fat synthesis and energy reserve by increasing the absorption of energy from the diet, altering the duration of intestinal digestion and absorption, or by regulating signalling pathways. It was found that obese people have abnormal gut microbiota composition and metabolism of short-chain fatty acids (SCFAs) [12]. Ley et al. [15] found significant differences in the structure of distal colon flora between obese and non-obese people in a controlled study of 12 subjects. The proportion of *Firmicutes* in the obese group was like that of the non-obese group after 1 year of body mass reduction. The physiological, biochemical and metabolic changes of the tissues and organs in women during pregnancy are obvious, and these changes are accompanied by changes in the colonised flora. The gut microbiota of older pregnant women differed significantly from that of women who were of normal reproductive age, with the diversity of the gut microbiota decreasing as gestational age increased; more *Firmicutes* and *Clostridium* were present in early pregnancy and an increase in *Enterobacterium* and *Streptococcus* were seen in late pregnancy [16]. The study by Santacruz et al. [17] showed that the contents of *Staphylococcus*, *Enterobacteriaceae*, and *E. coli* were significantly higher in obese (*n* = 16) and normal weight pregnant women (*n* = 34) than in normal weight pregnant women at 24 weeks of gestation, while the contents of Bifidobacterium and Bacteroidetes were lower. The differences in gut microbiota between obese and normal weight pregnant women are well established. Studies have shown that excessive weight gain during pregnancy is closely related to the level of serum SCFAs [17]. Short-chain fatty acids are produced by the fermentation of food by intestinal microorganisms (e.g. propionic acid, butyric acid and acetic acid). Increased levels of SCFAs, especially propionate and butyrate, are important factors contributing to abnormal metabolism and low inflammatory status [16]. It has been hypothesised that abnormalities of gut microbiota during pregnancy may affect maternal–foetal metabolism through SCFAs [9]. In addition, the addition of probiotics during pregnancy can improve glucose tolerance and insulin sensitivity in pregnant women; once again, this indicates the influence of gut microbiota on metabolism during pregnancy [18].

We found that when maternal EGWG occurred, neonatal Alpha diversity was lower in the prepregnancy overweight/obese group compared with the prepregnancy normal group, and there was a statistically significant difference in Beta diversity between PBMI and GWG (N-EG and N-NG group; N-EG and O-EG group). LEfSe analysis revealed that there were significant differences in microbiota abundance between newborns and pregnant women in the pre-pregnancy overweight/obese normal GWG maternal group. Aerobic anaerobic bacteria were increased in the gastrointestinal microflora of neonates in the EGWG group. Excessive increase of aerotolerant anaerobic bacteria in the intestinal tract of newborns will prevent or delay the maturation of gut microbiota. The abnormality of gut microbiota may affect maternal-fetal metabolism through SCFA, resulting in metabolic abnormalities and the appearance of low inflammatory state, causing harm to the parturient and fetus. Due to the short follow-up time, it is unclear whether newborns in the EGWG group will still have different gut microbiota from those in the GWG group in the future, and whether different gut microbiota will have an impact on the health of newborns in the EGWG group.

In summary, in this study we found that maternal weight gain during pregnancy impacts the composition of neonatal gut microbiota, and EGWG is likely to reduce alpha diversity and abundance of neonatal gut microbiota. However, some limitations remain in this study. Firstly, the molecular mechanisms by which maternal weight gain during pregnancy impacts the composition of the neonatal gut microbiota still need to be further explored. Secondly, the number of samples in this study is small, and more samples need to be collected later for further study. Finally, due to the limitation of sample size, some other influencing factors such as mode of delivery could not be analyzed, and subsequently, we will collect more samples for analyzing the association of maternal weight gain during pregnancy on neonatal gut microbiota composition.

## Conclusion

Maternal weight gain during pregnancy affects the composition of neonatal gut flora, and this study found that EGWG is likely to reduce the Alpha diversity and abundance of neonatal gut microbiota. Changes in the diversity and abundance of neonatal gut microbiota can lead to metabolic abnormalities and inflammation, which have an impact on neonatal health. Due to the small number of cases in this study and the lack of in-depth studies, multiple future experiments are needed to clarify the causal links and related mechanisms.

## Data Availability

The datasets generated and/or analysed during the current study are available in the Genome Sequence Archive (GSA) repository, [PRJCA011469].

## Abbreviations

N-NG group: Normal pre-pregnancy weight_normal pregnancy weight gain group
N-EG group: Normal pregnancy weight_excessive pregnancy weight gain group
O-NG group: Overweight/obese_normal pregnancy weight gain group
O-EG group: Overweight/obese_excessive pregnancy weight gain group
PBMI: Pregnancy body mass index
EGWG: Excessive gestational weight gain
IOM: Institute of Medicine
OTUs: Operational Taxonomic Units
SCFAs: Short-chain fatty acids
